# Accumulation of branched-chain amino acids deteriorates the neuroinflammatory response of Müller cells in diabetic retinopathy via leucine/Sestrin2-mediated sensing of mTOR signaling

**DOI:** 10.1007/s00592-024-02349-3

**Published:** 2024-08-16

**Authors:** Qiaoyun Gong, Jingyi Wang, Dawei Luo, Yupeng Xu, Rulin Zhang, Xin Li, Zihan Yin, Junwei Fang, Haiyan Wang

**Affiliations:** 1 Department of Ophthalmology, Shanghai General Hospital, Shanghai Jiao Tong University, Shanghai, China; 2 National Clinical Research Center for Eye Diseases, No. 100 Haining Road, Hongkou District, Shanghai, 200080 China; 3 Shanghai Key Laboratory of Ocular Fundus Diseases, Shanghai, China; 4 Shanghai Engineering Center for Visual Science and Photomedicine, Shanghai, China; 5 Shanghai Engineering Center for Precise Diagnosis and Treatment of Eye Disease, Shanghai, China; 6 Department of Laboratory Medicine, Shanghai General Hospital, Shanghai, China; 7 Department of Ultrasound, Shanghai General Hospital, Shanghai Jiao Tong University School of Medicine, Shanghai, 200080 China

**Keywords:** Branched-chain amino acids, mTORC1, Inflammation, Glial, Diabetic retinopathy

## Abstract

**Aims:**

This study aimed to investigate branched-chain amino acid (BCAA) catabolism in diabetic retinopathy (DR).

**Methods:**

Wild-type and *db/db* mice were fed BCAAs (5 or 10 mg/kg/day) for 12 weeks, and hyperglycemia-exposed Müller cells were treated with BCAAs (2 or 5 mmol/L) for 24 and 48 h. BCAA levels were measured using MS/MS. Western blotting was performed to detect proteins. Flow cytometry, oxygen consumption rate, and Cell Counting Kit-8 assays were used to evaluate Müller cell viability. Each experiment was conducted at least thrice.

**Results:**

BCAAs and branched-chain α-keto acids (BCKAs) were increased in the retina and systemic tissues of diabetic mice, and these changes were further enhanced to approximately 2-fold by extra BCAAs compared to wild-type group. In vitro, BCAAs and BCKAs were induced in hyperglycemic Müller cells, and augmented by BCAA supplementation. The aberrant BCAA catabolism was accompanied by mTORC1 activation and subsequently induced TNF-ɑ, VEGFA, GS, and GFAP in retinas and Müller cells under diabetic conditions. The cell apoptosis rate increased by approximately 50%, and mitochondrial respiration was inhibited by hyperglycemia and BCAA in Müller cells. Additionally, mTORC1 signaling was activated by leucine in Müller cells. Knockdown of Sestrin2 or LeuRS significantly abolished the leucine-induced mTORC1 phosphorylation and protected Müller cell viability under diabetic conditions.

**Conclusions:**

We found that BCAA catabolism is hindered in DR through mTORC1 activation. Leucine plays a key role in inducing mTORC1 by sensing Sestrin2 in Müller cells. Targeting Sestrin2 may ameliorate the toxic effects of BCAA accumulation on Müller cells in DR.

## Background

Diabetic retinopathy (DR) is one of the main microvascular complications of diabetes mellitus and a leading cause of vision loss and blindness in working-age adults worldwide [1, 2]. Notably, DR is characterized by neovascularization and neurodegeneration, which interact with each other and cooperate to promote DR progression. Hyperglycemia is the primary cause of DR and induces pathophysiological fluxes, including inflammation, oxidative stress, apoptosis, and metabolic imbalance [1]. Moreover, early neurodegeneration in DR is considered an initiating factor of vascular damage [3], meanwhile, neovascularization in DR originates from the retinal vessels. The neuronal components of the retina are compromised in DR; in particular, Müller cells are polarized and retinal ganglion cells die early in DR [4, 5]. Functional studies of the retina under diabetic conditions supported observations made using electroretinography, dark adaptation, contrast sensitivity, and color vision tests, revealing that neuronal function decreased before the onset of vascular lesions in both humans and animal models of diabetes [6, 7]. The underlying reasons for early neuronal cell impairment in diabetic retina are not well understood. However, one study reported increased levels of neurodegenerative metabolites in diabetic retinas [8]. Glutamate is one of the major excitotoxic metabolites that is elevated in the vitreous and retina of patients with diabetes [9] and in animal models of diabetes [10] and may cause neuronal damage in the retina.

Circulating levels of branched-chain amino acids (BCAAs), including valine, leucine, and isoleucine, increase in individuals with obesity, insulin resistance (IR), and/or type 2 diabetes mellitus (T2DM) [11–13]. Furthermore, BCAA-related metabolites are predictive of type 2 diabetes pathogenesis and are prognostic markers of intervention outcomes [14, 15]. In our previous studies, elevated levels of BCAAs were detected by MS/MS analysis in the blood plasma, aqueous humor, and vitreous humor of patients with DR, as well as in the serum of *db/db* mice in vivo [16, 17]. In addition, a few studies have reported excess BCAAs in the serum and retina of diabetic rats [18, 19]. Therefore, high levels of BCAAs and related intermediate metabolites are not only biomarkers of type 2 diabetes but also pathophysiological factors involved in diabetic complications. However, few studies have explored how BCAAs regulate cellular homeostasis and affect DR progression. An analysis of changes in BCAAs and related metabolites in response to glucose challenge may provide a novel strategy for ameliorating neuroinflammation in early DR.

To explore the potential regulatory mechanisms of BCAAs associated with neurodegeneration, inflammation, and apoptosis in the diabetic retina, the process of BCAA catabolism was investigated. There are two main steps involved in BCAA metabolism [12]. First, BCAA transamination yields BCAA catabolites such as branched-chain α-keto acids (BCKAs), α-ketoisovalerate (KIC) from leucine, α-keto-β-methyl valeric acid (KMV) from isoleucine, and α-ketoisobutyrate (KIV) from valine. This reversible step is mediated by branched-chain aminotransferases (BCATs, BCAT1 and BCAT2). Second, irreversible initiation of BCKA oxidation occurs via the BCKA dehydrogenase (BCKDH) complex encoded by the BCKDHA and BCKDHB genes. Furthermore, BCKD activity is regulated by posttranslational modifications of phosphorylation. Phosphorylation of BCKD E1α subunit by BCKD kinase (BCKDK) inhibits BCKD, whereas BCKD dephosphorylation by the protein phosphatase 2Cm activates it. These three BCAAs differentially stimulate the protein kinase mechanistic target of rapamycin complex 1 (mTORC1), a key regulator of many metabolic processes, and are involved in the posttranslational modification of distinct proteins. Although the catabolism of each BCAA involves several steps, the intermediate and final products are distinct. Overall, the steps and enzymes involved in BCAA catabolism are closely associated with the accumulation of BCAAs and BCKAs, thus affecting their toxic effects in DR.

This study aimed to assess whether excess BCAAs can further impair BCAA catabolism and neuroinflammation in Müller cells under hyperglycemic conditions. We hypothesized that BCAA catabolism was hindered under diabetic condition to induce the accumulation of BCAAs and BCKAs, which caused the stimulation of neuroinflammation in Müller cells. The individual BCAA might played a key role in activating mTORC1 signaling to affect Müller cell function in DR. This study would also identify the dominant amino acids involved in BCAA catabolism during early DR.

## Methods

### Experimental animal study

Seven-week-old male wild-type (WT) and diabetic *db/db* mice were obtained from the Animal Research Center of Shanghai General Hospital. The genetic background of the *db/db* mice was C57BL/KsJ (BKS). This study was approved by the Ethics Committee of Shanghai General Hospital and performed in accordance with the National Institutes of Health Guide for the Care and Use of Laboratory Animals. All experimental mice were maintained under specific pathogen-free conditions with a 12/12 h light/dark cycle at 22 ± 2 °C in an animal facility. After 1 week of acclimatization, the WT and diabetic mice were randomized into different treatment groups, namely, the non-BCAA-fed (control, ctr), low dose BCAAs-fed (5 mg/kg/day), and high dose-BCAAs fed (10 mg/kg/day) groups, through intragastric administration. Each group contained eight mice. The WT and *db/db* mice were gavaged with equal amounts of 0.9% saline solution. After 12 weeks, the mice were sacrificed by exsanguination under anesthesia by inhalation of 5% isoflurane in ambient air. The eyeballs were extracted from each group to prepare retinal tissues. Retinal tissues were separated for protein quantification and mass spectrometry (MS) analysis. The plasma, kidney, liver, and heart samples were collected for MS analysis.

### Human retinal Müller cell culture and treatment

Moorfields/Institute of Ophthalmology-Müller 1 (MIO-M1) cells were maintained in Dulbecco’s modified Eagle’s medium/F12 (Gibco, Waltham, MA, USA) supplemented with 10% fetal bovine serum at 37 °C in a 5% CO_2_ atmosphere. The cell passage of 3–5 was used in this study. Cultures of Müller cells at 70–80% confluence were exposed to 5.5 mmol/L D-glucose (normal glucose, NG), 25 mmol/L D-glucose (high glucose, HG), HG without BCAAs, HG + low BCAAs (2 mmol/L), or HG + high BCAAs (5 mmol/L) for 24–48 h. Furthermore, BCAAs were prepared by mixing L-leucine, L-isoleucine, and L-valine at a ratio of 2:1:1.2, and the molecular weight was 126.829 g/mol [20]. Individual BCAAs were supplemented with 5 mmol/L leucine, isoleucine, or valine in HG medium.

### Transfection with small interfering RNA (siRNA)

After reaching 70-80% confluence, Müller cells were transfected with Sestrin1, Sestrin2, leucyl-tRNA synthetase (LeuRS), or negative control (NC) siRNAs using Lipofectamine RNAiMAX transfection reagent (Invitrogen, Carlsbad, CA, USA). Notably, 5 h after transfection, the medium was replaced with a fresh medium. After 48 h of transfection, the cells were harvested for protein analysis. Sestrin1, Sestrin2, LeuRS, and NC siRNAs were synthesized by GenePharma (Shanghai, China).

### Western blot analysis

Proteins were isolated from mice retinal tissues or cultured Müller cells with lysis buffer (Beyotime, #P0013B, China) supplemented with PMSF (Sigma, #ST506, USA) and phosphatase inhibitor (Beyotime, #S1873, China). Protein concentrations were determined using a Bicinchoninic Acid Protein Assay Kit (Beyotime, Jiangsu, China). Proteins were denatured, separated by SDS‒PAGE, and transferred to PVDF membranes. After blocking with 5% nonfat milk, the membranes were incubated with the following primary antibodies diluted 1:1000 overnight at 4 °C: BCAT1 (Abcam, ab232706, USA), BCAT2 (Abcam, ab95976, USA), BCKDK (Abcam, ab151297, USA), BCKDHA (Abcam, ab200577, USA), BCKDHB (Abcam, ab201225, USA), TNF-α (CST, #11948S, USA), VEGFA (Abcam, ab214424, USA), S6 (CST, #2217S, USA), p-S6 (CST, #4858S, USA), p70 s6k (CST, #34475, USA), p-p70 s6k (CST, #9234, USA), GFAP (CST, #12389, USA), GS (Abcam, ab313449, USA), and GAPDH (1:2000, CST, #5174, USA) or β-actin (CMC-TAG, AT0001, USA). After washing with TBST buffer, the membranes were incubated with an HRP–conjugated secondary antibody containing anti-rabbit (1:5000, CST, #7074, USA) or anti-mouse IgG (1:5000, CST, #7076, USA) for 2 h at 37 °C. Finally, the protein bands were visualized using an enhanced chemiluminescence (ECL) kit (Thermo, #NCI5079, USA). GAPDH or β-actin was used as the loading control. Each band was quantified using the ImageJ software.

### Flow cytometry

Müller cells from different treatment groups were harvested for apoptosis analysis using an APC Annexin V kit (#561012, BD Biosciences, Franklin Lakes, NJ, USA). In brief, 1 × 10^5^ Müller cells were collected and incubated with Annexin V and propidium iodide. The percentage of apoptotic cells was determined using a FACSAria flow cytometer (BD Biosciences).

### Cell counting Kit-8 (CCK-8) assay

Cell viability was measured using a CCK-8 assay kit (#E606335, Sangon Biotech, Shanghai, China) according to the manufacturer’s protocols. Müller cells were cultured and exposed to different culture conditions and then plated in 96-well plates at a density of 2 × 10^4^ cells/well for 48 h. Subsequently, the medium was changed with 100 µL of fresh medium and 10 µL of CCK-8 reagent was added to each well. After incubation for 1 h, the absorbance was measured at 450 nm using a Varioskan Flash multimode reader (Thermo Fisher Scientific).

### UHPLC-MS/MS analysis of BCAAs

The retinal tissue, kidney, liver, heart, and blood plasma samples were collected and processed for UHPLC-MS/MS analysis. The analysis was performed using a hybrid quadrupole-Orbitrap mass spectrometer (Q Exactive, Thermo Scientific, Bremen, Germany) coupled to an HPLC system equipped with qualified settings. Chromatographic separation of amino acids was achieved by reverse-phase (RP) analysis on a 150 mm × 2.1 mm C18 (2.6 μm 100 Å) (Agilent, USA) under the following conditions: solvent A was water with 0.1% formic acid, and solvent B was acetonitrile with 0.1% formic acid. The separation gradient was initially 2% B, maintained for 2 min, then increased linearly from 2 to 100% B in 10 min, washed with 100% B for 3 min, and column equilibration with 2% B for 3 min. The total run time was 18 min, with a flow rate of 0.25 mL/min and an injection volume of 1 µL. A small-scale database was constructed using the BCAA/BCKA names, retention times (RT), and accurate mass-to-charge ratios (m/z). Next, automated peak picking, integration, RT adjustment using the aforementioned database, and alignment were performed using the open-source software Mzmine. Data were extracted from Mzmine using the following parameters: alignment (join aligner m/z tolerance, 1 ppm; RT tolerance, 30 s; weight for m/z, 15; and RT, 10).

Müller cell samples were processed for liquid chromatography-MS (LC-MS) analysis. Subsequently, LC-MS was performed using an Agilent 1290 UHPLC system equipped with a binary solvent delivery manager coupled to a Triple QTRAP 6500 + System (AB Sciex, Framingham, MA, USA). The cell samples were extracted with protein precipitation and separated on a 1.8 μm (2.1 × 100 mm) column (ACQUITY UPLC ^®^ HSS T3, Wates) through HPLC. Each mass response was corrected with the response of the respective internal standard (leucine and isoleucine were corrected using L-leucine-d3 and valine was corrected using L-valine-d8; Cambridge Isotope Laboratories, MA). Each peak area ratio was used for relative quantitation. The data were processed using OS V 2.1 software (AB SCIEX).

### Measurement of mitochondrial respiration by a Seahorse system (oxygen consumption rate [OCR])

Mitochondrial respiration was determined by measuring OCR using a Seahorse XF analyzer (Agilent Technologies, Santa Clara, CA, USA). Müller cells seeded in 24-well microplates at a density of 20,000 cells per well were incubated under different experimental conditions and washed 2× with assay medium (Seahorse XF DMEM supplemented with 1 mM sodium pyruvate, 2 mM glutamine and 10 mM glucose). Subsequently, 1 mL of assay medium was added in each well and incubated at 37 °C for 30 min. The OCR was determined using a Seahorse XF Cell Mito Stress Test Kit (cat. no. 103015-100, Agilent Technologies) according to the manufacturer’s instructions. For OCR determination, the ATP synthesis blocker oligomycin (1.5 µM), a mitochondrial uncoupler, carbonyl cyanide p-trifluoro methoxyphenylhydrazone (2.0 µM FCCP), and inhibitors of complex I and complex III, rotenone/antimycin A (0.5 µM) were injected in ports A, B and C, respectively. The values of OCR reflect the metabolic activities of the cells and the total number of cells alive, so oxygen consumption and extracellular acidification rates were normalized to the total amount of cells in each well. The cell number was relatively quantified by detecting the protein concentrations using a Bicinchoninic Acid Protein Assay Kit (Beyotime, Jiangsu, China). Basal respiration, maximal respiration, spare respiratory capacity, and ATP production were measured, normalized to cell numbers, and analyzed using Wave software (Agilent Technologies).

### Statistical analysis

Statistical analyses were conducted using GraphPad Prism 7.0 software. All data are presented as the mean ± SEM, and the sample size is indicated with the corresponding results. One-way ANOVA followed by Tukey’s test was used for comparisons among multiple groups, and the unpaired Student’s t-test was used for comparisons between two groups. Statistical significance was set at *p* < 0.05.

## Results

### BCAA accumulation characterizes BCAA metabolism deficiency in diabetic mice and Müller cells

**Fig. 1:**
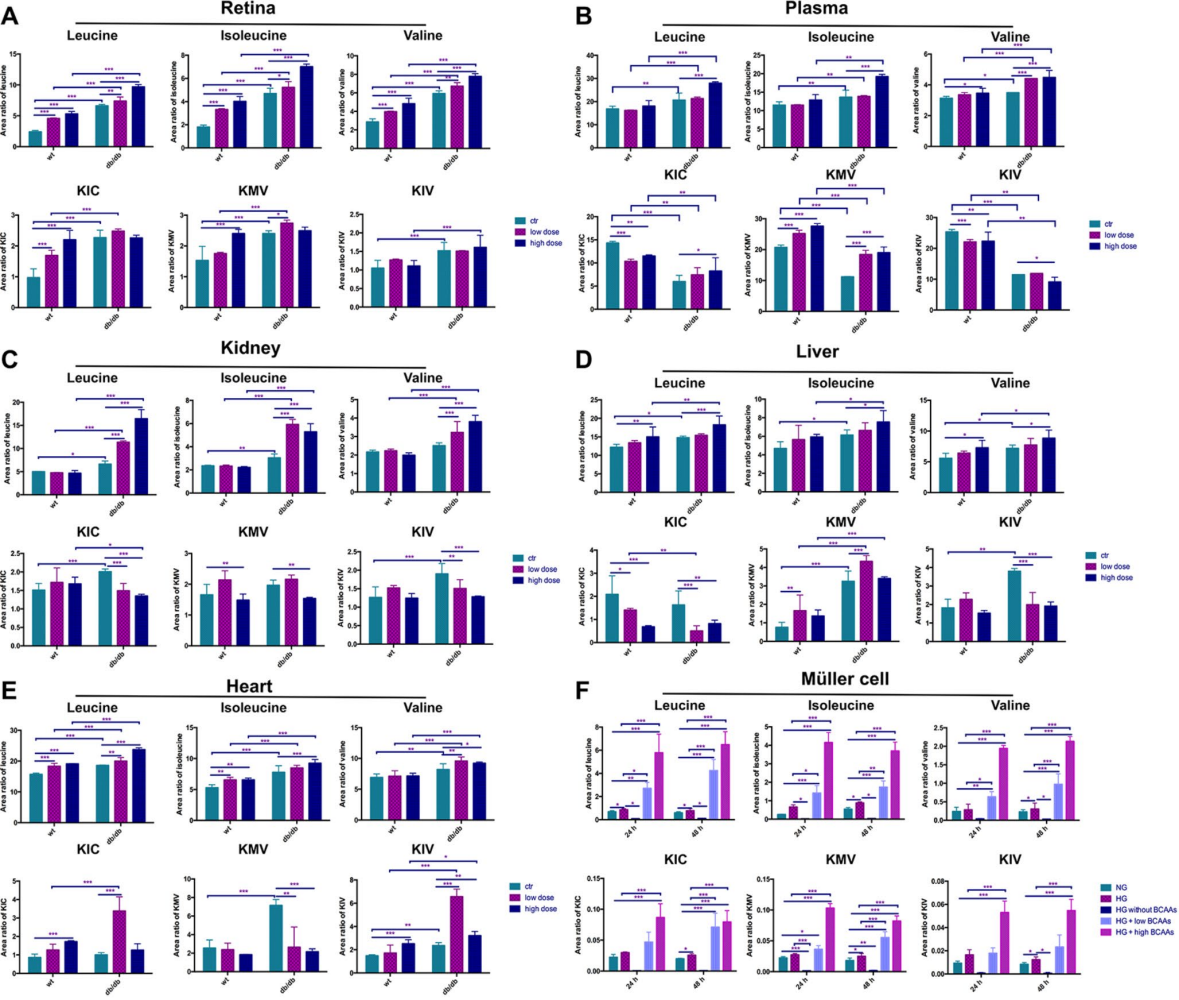
Accumulated BCAAs and BCKAs in *db/db* mice fed with BCAAs and Müller cells. (**A**) The levels of BCAAs (leucine, isoleucine and valine) and BCKAs (KIC, KMV and KIV) were greater in the retinas of *db/db* mice than in those of wt mice. Food feeding with extra BCAAs further aggravated the high levels of BCAAs and BCKAs. The levels of BCAAs and BCKAs detected systemically in the plasma (**B**), kidney (**C**), liver (**D**) and heart (**E**). (**F**) In vitro, BCAAs and BCKAs were more abundant in high glucose-cultured Müller cells than in normal glucose-cultured Müller cells. The deprivation of BCAAs in high-glucose medium significantly suppressed the levels of BCAAs and BCKAs. The addition of a low dose or high dose of BCAAs for 24–48 h distinctly induced the accumulation of BCAAs and BCKAs. wt, wild type; *db/db*, diabetic mice; *p < 0.05, **p < 0.01, ***p < 0.001; n = 3

Shown by our previous study [16], db/db mice used from eight-week old, and sustained for another 12 weeks with different treatments, reflected the early stage of diabetic retinopathy. Similar to the clinical changes in DR in the early stage, microaneurysm, exudation, and hypoperfusion were observed in db/db mice by fundus fluorescent angiography. This hinted us to clarify the essential target to ameliorate DR before angiogenesis and neurodegeneration. Therefore, to investigate the abundance of BCAAs in retinas and other tissues from BCAA-fed WT and db/db mice, LC-MS/MS analysis was performed. Three BCAAs (leucine, isoleucine, and valine) and BCKAs (KIV, KIC, and KMV) were separately quantified in the retinal tissue, plasma, kidney, liver, and heart of each group. BCAA levels in retinal tissues were significantly elevated in *db/db* mice compared to WT mice (Fig. 1A). In both WT and *db/db* mice, BCAA administration increased leucine, isoleucine, and valine in response to increasing doses. Moreover, BCAA accumulation in the diabetic retina was further augmented than that in the WT group. Furthermore, KIC, KMV, and KIV were enhanced and augmented by BCAA administration in the retinas of db/db mice than those of WT mice. Notably, BCAAs cannot be endogenously synthesized in animals and are only acquired from food sources. To evaluate the catabolic alteration of BCAAs by additive feeding in diabetic mice, we conducted a targeted metabolomic analysis to characterize BCAA catabolism in *db/db* mice by detecting the abundance of BCAAs and their metabolites in plasma and various tissues. As expected, the plasma BCAA levels were higher in *db/db* mice, especially in those fed BCAAs (Fig. 1B). Furthermore, MS revealed similar specific measurements of plasma BCAA levels in the kidney, liver, and heart (Fig. 1C–E). Gene expression analysis suggested the systemic downregulation of the BCAA catabolic pathway under diabetic conditions. In contrast, the plasma BCKA concentrations were significantly lower in *db/db* mice than in WT mice. In WT mice, BCAA administration distinctly enhanced KMV levels in a dose-dependent manner; however, it suppressed KIC and KIV levels. Whereas, in diabetic mice, supplementation with a high dose of BCAAs increased KIC and KMV levels. Notably, KIC and KIV in the kidneys and KMV and KIV in the liver and heart were significantly higher in diabetic mice than in WT mice. However, BCAA administration increased BCKA levels in WT mice and decreased BCKA levels in *db/db* mice. Therefore, BCAA catabolism was suppressed at the pathway level in various tissues of *db/db* mice, accompanied by reduced catabolic flux and BCAA accumulation.

To evaluate BCAA catabolism in Müller cells under diabetic conditions in vitro, MS analysis was performed to detect BCAA and BCKA levels. Exposure to high glucose for 48 h significantly increased BCAA and BCKA levels (Fig. 1F). As expected, BCAA deprivation from high-glucose media suppressed BCAA and BCKA levels in Müller cells. In contrast, further supplementation of the high-glucose media with BCAAs augmented the accumulation of BCAAs and BCKAs. This observation is consistent with the observed alterations in BCAA catabolism in diabetic retinas. Therefore, the regulation of BCAA catabolic flux by rate-limiting enzymes in diabetic retinas and retinal cells requires further investigation.

### BCAA catabolic deficiency induced inflammation, vascular damage, and gliosis of Müller cells via mTORC1 in *db/db* mice

**Fig. 2:**
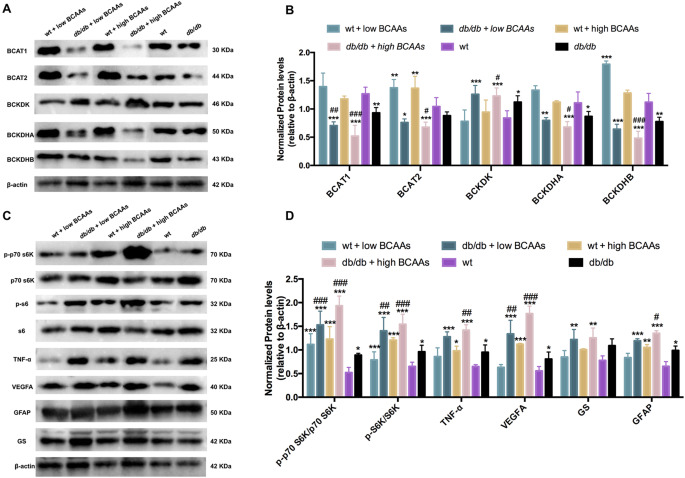
BCAA catabolism affected inflammation, neovascularization and glial activation in Müller cells via mTORC1 signaling in vivo. (**A**) The grey blots of enzymes involved in BCAA catabolism in the retinas of *db/db* mice fed with different doses of BCAAs. (**B**) Quantification of blots by ImageJ showed that BCAT1, BCAT2, BCKDHA and BCKDHB were inhibited in diabetic retinas and deteriorated by BCAAs supplementation, while BCKDK demonstrated the opposite changes in expression. (**C**) Western blots showing the phosphorylation of p70 S6K and S6K and TNF-α, VEGFA, GFAP and GS in the retinas. (**D**) Phosphorylation of p70 S6K and S6K was enhanced in the retinas of *db/db* mice and further increased by BCAAs feeding. The levels of TNF-α, VEGFA, GFAP and GS increased with BCAAs accumulation in diabetic retinas. wt, wild type; *db/db*, diabetic mice; *p < 0.05, **p < 0.01, ***p < 0.001, compared to the wt group; ^#^*p* < 0.0, ^##^*p* < 0.01, ^###^*p* < 0.00, compared to the db/db group; *n* = 3. β-actin was used as the reference gene

The accumulation of BCAAs and BCKAs in the diabetic retina suggested that BCAA catabolism was blocked. Therefore, the expression of genes encoding enzymes involved in the first and second steps of BCAA metabolism was analyzed. Notably, *db/db* and WT mice were fed low- or high-dose BCAAs for 12 weeks. In the first reversible step, BCAT1 and BCAT2 levels decreased in the retinas of *db/db* mice. Furthermore, *BCAA administration* in *db/db* mice did not rescue the downregulation of BCAT1 and BCAT2; however, it aggravated the decrease in BCAT1 and BCAT2 expression in the retina (Fig. 2A, B). In WT mice, BCAA administration activated BCAT1, although the difference was not significant compared to that in WT mice. However, BCAT2 expression increased with the addition of low- or high-dose BCAAs compared to that in WT mice. This suggests that mitochondrial metabolism is activated by BCAA addition in WT mice under physiological conditions. Further analysis of the second irreversible step revealed that BCKDHA and BCKDHB were downregulated and BCKDK was elevated in the retinas of *db/db* mice treated with or without BCAAs (Fig. 2A, B). This was more obvious in the high-dose group. In the retinas of WT mice, BCKDHB was significantly increased under a low dose of BCAAs, whereas BCKDK did not demonstrate significant changes after *BCAA administration* compared with the WT group. These results revealed that under physiological conditions, BCAA catabolism can be activated by BCAA addition, whereas diabetes obstructs BCAA catabolism, and BCAA supplementation exacerbates this obstruction in *db/db* mice.

Growing evidence suggests that BCAA metabolism is involved in the regulation of several pathophysiological processes, including inflammation and neovascularization. In this study, we evaluated the modulatory roles of BCAAs in these essential pathophysiological processes in the diabetic retina. Unsurprisingly, under diabetic conditions, inflammatory and angiogenic responses were induced in the retina than in the WT group. With *BCAA administration*, WT and *db/db* mice exhibited increased TNF-α and VEGFA levels (Fig. 2C, D), with changes in the *db/db* mice being more significant. Müller cells play a crucial role in maintaining retinal neuronal homeostasis and in providing support and protection to neurons. Under pathological conditions such as DR, Müller cells undergo reactivation. As shown in this study, GFAP and GS levels were enhanced in the retinas of diabetic mice. Supplementation with BCAAs further increased GFAP and GS levels in diabetic retinas (Fig. 2C, D). This suggests that Müller cell activation is induced by diabetes and is deteriorated by BCAAs. Furthermore, BCAAs enhance mTORC1 activity. This study showed that BCAA intake significantly upregulated mTORC1 activation in the retinas of both WT and *db/db* mice. Compared to the WT group, the phosphorylation of p70 S6K and S6K in diabetic retinas increased 2-fold. *BCAA administration* enhanced p-p70 S6K/p70 S6K by nearly 2-fold in response to a low dose of BCAAs and 3-fold in response to a high dose of BCAAs in the WT and *db/db* group. Therefore, BCAA intake impairs defective BCAA catabolic flux by enhancing mTORC1 activity in diabetic retinas.

### Excess BCAAs/BCKAs contributed to the cellular dysfunction of Müller cells under hyperglycemic conditions

**Fig. 3:**
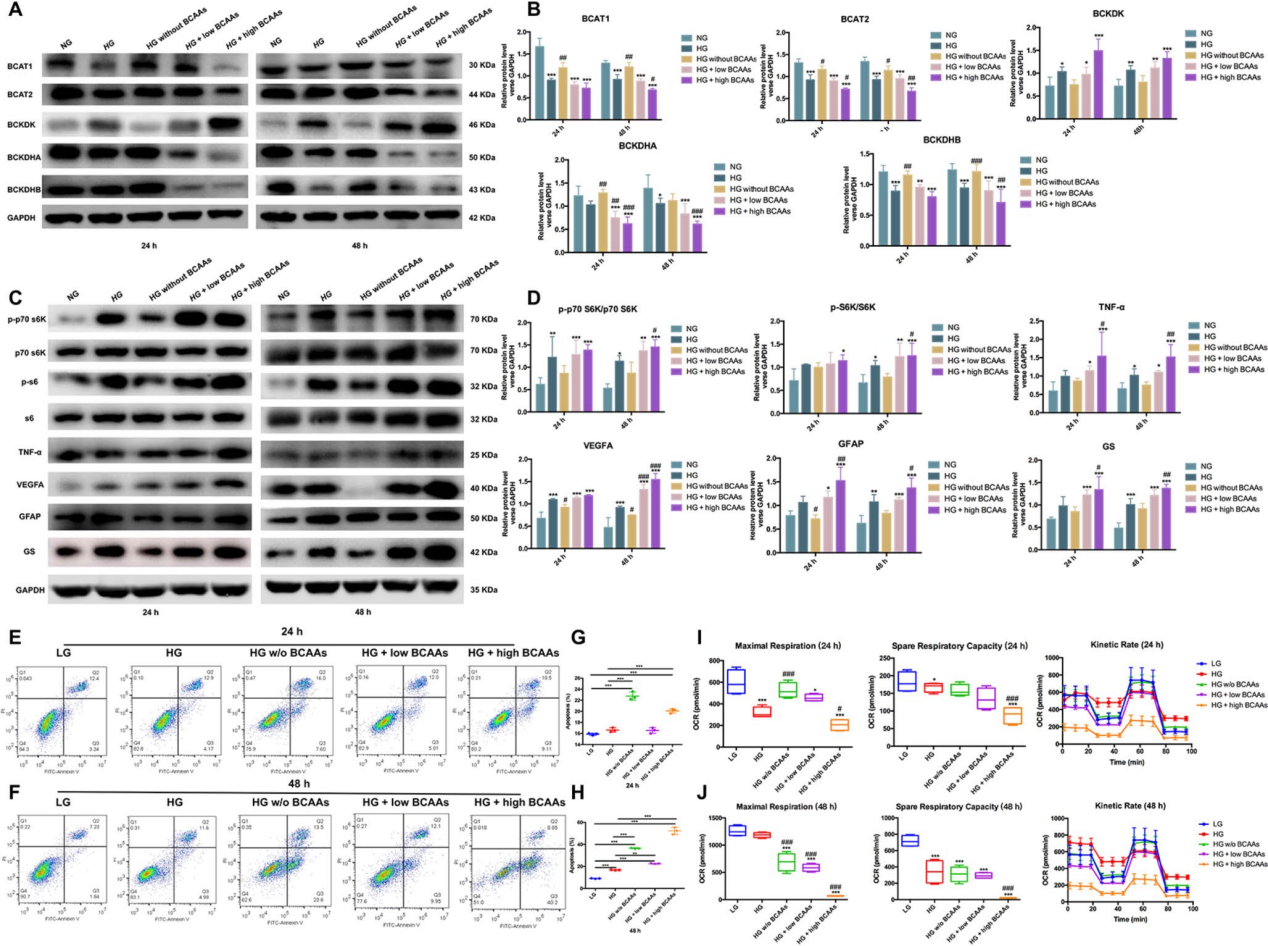
Impaired BCAA catabolism contributed to cellular dysfunction in Müller cells under hyperglycemic conditions in vitro. (**A**) The grey blots of the BCAA catabolic enzymes in Müller cells treated with different doses of BCAAs under high glucose conditions for 24 h and 48 h. (**B**) Quantification of blots revealed that the protein levels of BCAT1, BCAT2, BCKDHA and BCKDHB were decreased in hyperglycemic cells and further inhibited by BCAAs addition, while BCKDK was enhanced significantly. (**C**, **D**) The expression levels of p-p70 S6K/p70 S6K, p-S6K/S6K, TNF-α, VEGFA, GFAP and GS were elevated by high glucose and BCAAs supplementation in Müller cells cultured for 24–48 h. (**E**-**H**) Müller cells were collected. The apoptotic rate and statistical analysis of Müller cells treated with different doses of BCAAs under HG conditions. (**I**, **J**) Mitochondrial dysfunction caused by high glucose and BCAAs accumulation in Müller cells was revealed by OCR and ATP production analysis (*n* = 4). *p < 0.05, **p < 0.01, ***p < 0.00, compared to the NG group; ^#^*p* < 0.0, ^##^*p* < 0.01, ^###^*p* < 0.001, compared to the HG group; *n* = 3. GAPDH was used as the reference gene

We conducted in vitro experiments in Müller cells under hyperglycemic conditions to explore the underlying mechanisms of BCAA catabolism. In Müller cells cultured under hyperglycemic conditions, BCAA catabolism showed a profile similar to that observed in vivo. BCAAs and BCKAs were induced and accumulated under hyperglycemic conditions in Müller cells. These parameters were significantly enhanced with the addition of BCAAs. When the high-glucose medium was not treated with BCAAs, these levels decreased (Fig. 3A, B). The levels of BCAAs and BCKAs were found to change significantly, especially after 48 h of treatment. The results revealed that the initial oxidation of BCAAs was hindered by the reduction of BCAT1 and BCAT2 under hyperglycemic conditions and was decreased by BCAA supplementation. Furthermore, BCKDHA and BCKDHB were decreased, whereas BCKDK was enhanced in high glucose-treated Müller cells. Additionally, BCAA catabolic defects caused by high glucose levels were augmented by BCAAs treatment. Subsequently, we observed alterations in mTORC1 activity and subsequent inflammation, angiogenesis, and reactivation of Müller cells after *BCAA administration*. As expected, BCAA accumulation enhanced the phosphorylation of mTORC1. Furthermore, p-p70 S6K/p70 S6K and p-S6K/S6K increased significantly in hyperglycemia-treated Müller cells (Fig. 3C, D). The addition of a high dose of BCAAs for 48 h further enhanced the p-p70 S6K/p70 S6K and p-S6K/S6K levels. Moreover, similar increases in TNF-α and VEGFA were observed in Müller cells treated with high glucose and *BCAA administration*. In addition, activation of GFAP and GS was found in Müller cells exposed to hyperglycemia. A high dose of BCAAs further enhanced the expression of GFAP and GS in Müller cells under hyperglycemic conditions. These results supported that BCAA and BCKA accumulation induced by high glucose stimulated mTORC1, thus promoting subsequent inflammatory and angiogenic signaling and Müller cell reactivation.

To examine the effects of BCAA catabolic defects on Müller cells under diabetic conditions, flow cytometry was used to evaluate apoptosis, and mitochondrial respiratory activity was analyzed. In the context of hyperglycemia, Müller cell apoptosis increased and was further enhanced by the addition of BCAAs, especially at a high dose for 48 h. Unexpectedly, BCAA deprivation accelerated the apoptosis rate of Müller cells compared to either normal or high-glucose conditions. This finding suggests that normal levels of BCAAs are necessary to maintain cellular homeostasis (Fig. 3E–H). High glucose exposure for 24–48 h inhibited maximal expiration and spare-respiratory capacity, as revealed by the observed changes in OCR (Fig. 3I, J). After supplementation with low- or high-dose BCAAs for 48 h, the mitochondrial respiratory capacity was significantly lower than that under normal or high glucose conditions. When the high-glucose medium was deprived of BCAAs for 48 h, maximal expiration and spare-respiratory capacity decreased. A kinetic study of the ATP production rate demonstrated that hyperglycemic cells exhibited a higher ATP production rate associated with glycolysis than control cells. However, when Müller cells were supplemented with BCAAs, the ATP production rate was significantly inhibited. These results indicated that high glucose disrupted the respiratory capacity of mitochondria, and a baseline of BCAAs supported the mitochondrial function of Müller cells.

### Leucine was the dominant amino acid that activated mTORC1 signaling via the Sestrin2 sensor/LeuRS in Müller cells under hyperglycemic conditions

**Fig. 4:**
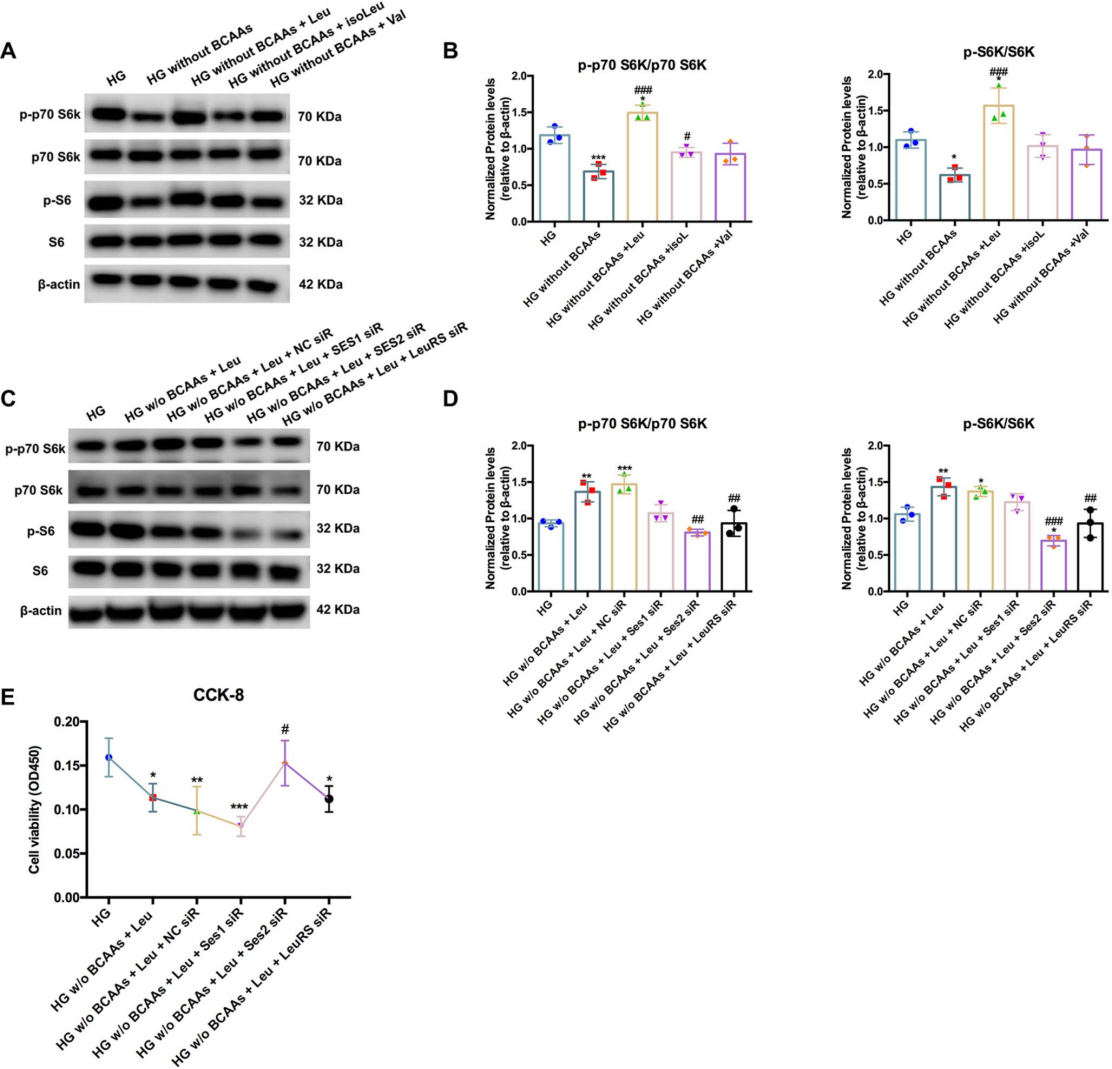
Leucine activated mTORC1 signaling via Sestrin2/LeuRS in Müller cells under hyperglycemia. (**A, B**) Western blot experiments demonstrated that the phosphorylation of mTORC1 was significantly increased by leucine in Müller cells under hyperglycemia without complicated BCAAs. (**C, D**) Knockdown of Sestrin2 or LeuRS suppressed the stimulation of the mTORC1 complex, including p70 S6K and S6K, with leucine overexpression in Müller cells under high glucose conditions. (**E**) Sestrin2 interference protected cell viability from leucine-induced damage to Müller cells under HG conditions. *p < 0.05, **p < 0.01, ***p < 0.001, compared to the HG group; ^#^*p* < 0.05, ^##^*p* < 0.0, ^###^*p* < 0.00, compared to the HG without BCAAs group for A and B, compared to the HG without BCAAs added leucine group for C and D; *n* = 3. β-actin was set as the reference gene

According to the in vivo and in vitro results, BCAA accumulation under diabetic conditions activates mTORC1 signaling. To further clarify the dominant amino acid of BCAAs enhancing mTORC1 signaling in Müller cells under high glucose conditions, separate amino acid was added. Upon BCAA starvation in a high-glucose medium, phosphorylation of the mTORC1 substrates p70 S6K and S6K was inhibited. However, when Müller cells were cultured with leucine added in high glucose without BCAAs, the phosphorylation of mTORC1 was significantly enhanced. Isoleucine alone only increased p-p70 S6K/p70 S6K in cultured Müller cells under hyperglycemic conditions deprived of BCAAs. Supplementation with valine alone did not affect the activation of mTORC1 (Fig. 4A, B). In cultured cells, mTORC1 senses leucine through leucine-binding Sestrin proteins, including Sestrin1 and Sestrin2. To determine whether mTORC1 regulation by leucine requires Sestrin1 and Sestrin2, we generated siRNAs targeting Sestrin1/Sestrin2 in Müller cells cultured with high glucose supplemented with only leucine. In addition, LeuRS plays a crucial role in amino acid-induced mTORC1 activation by sensing intracellular leucine concentration and initiating molecular events that cause mTORC1 activation. Therefore, we performed an interference experiment, in which LeuRS was knocked down. As shown by western blotting, loss of Sestrin1 did not affect the response of mTORC1 to leucine addition, which was enhanced in Müller cells cultured under hyperglycemic conditions (Fig. 4C, D). However, Sestrin2 knockdown reduced leucine sensing and significantly inhibited the activation of mTORC1 signaling. These results suggested that Sestrin2 plays a critical role in sensing leucine to regulate mTORC1 activation in vitro under diabetic conditions. In addition, reducing LeuRS levels by siRNA transfection suppressed leucine-induced activation of mTORC1 compared to that in HG medium containing only leucine (Fig. 4C, D). Transfection with the NC siRNA did not affect the response of mTORC1 to leucine. Therefore, leucine activated the phosphorylation of the mTORC1 complex by sensing Sestrin2 and binding to LeuRS in Müller cells under hyperglycemic conditions. After reversing the increase in mTORC1 expression by knockdown of Sestrin2 or LeuRS, the viability of Müller cells improved under hyperglycemic conditions (Fig. 4E). Taken together, these results established that leucine activated the mTORC1 signaling pathway by sensing Sestrin2 and binding to LeuRS in hyperglycemia-induced Müller cells.

## Discussion

DR is one of the most common microvascular complications associated with diabetes mellitus. Initially, DR is caused by long-term detrimental hyperglycemia and progresses to retinal inflammation, oxidative stress, apoptosis, neurodegeneration, and neovascularization [1]. Evidence that amino acids play a role in inflammation and neurodegeneration during DR development is based on measurements of elevated BCAAs in the vitreous and plasma of patients with diabetes [17, 21], in addition to the altered BCAA metabolism found in diabetic retinas and cells [16, 22]. This study explored the critical role of disrupted BCAA *catabolism* in DR development and the therapeutic potential of targeting the predominant BCAA sensor.

Notably, BCAAs are essential amino acids that are derived from protein-containing foods. A balanced BCAA homeostasis is essential for protein synthesis and energy production. Additionally, BCAAs act as signaling molecules that regulate glucose metabolism, inflammation, oxidative stress, and protein metabolism, thus affecting metabolic crosstalk in metabolic diseases in mammals [23]. However, in diabetes and diabetic complications, disrupted BCAA *catabolism* significantly breaks the physiological functions of different tissues. Furthermore, BCAA accumulation in the plasma is strongly associated with insulin resistance in individuals with obesity and T2DM [24, 25]. Additionally, in the present study, BCAAs levels increased in the plasma of *db/db* mice. Supplementation with BCAAs further aggravated BCAA accumulation in the plasma. To investigate the underlying cause, an observational and community-based study was conducted by Phielix et al., which provided evidence that higher plasma BCAA levels might originate partly from low mitochondrial oxidative capacity in patients with T2DM [24]. Elevated BCAA levels are associated with numerous systemic disorders of the muscle, brown fat, liver, kidneys, and heart. In diabetic mice subjected to MI/R injury, the levels of cardiac BCAAs and its metabolite BCKAs are increased, which supports defects in BCAA catabolism [26]. Similarly, *results from LC/MS performance revealed* that BCAAs and BCKAs levels were increased in the hearts of *db/db* mice, and *BCAA administration* significantly enhanced this increase. Analysis of the tissue distribution of BCAA disposal at the whole-body level revealed that the liver is one of the largest contributors to BCAA disposal into protein synthesis [23]. Therefore, detecting BCAA levels in the liver is important to predict BCAA transportation and metabolism systemically. As reflected by the MS results in this study, BCAA levels were elevated in the liver, and with high-dose BCAA administration, the increase in BCAA levels was further aggravated. Diabetic kidney disease (DKD) is another common microvascular complication of diabetes. Serum BCAA levels were significantly elevated in patients with T2DM compared to healthy individuals. However, these levels gradually decrease with DKD progression. Serum BCAA levels are independently correlated with renal function indicators [27]. In addition, the kidneys have abundant BCAA oxidation flux [23]. Under steady-state homeostasis, the BCAAs released from proteins must be either oxidized or reused for protein synthesis. Therefore, the kidney is essential for inducing BCAA oxidation flux to maintain homeostasis. In *db/db* mice, we found that BCAA levels were significantly increased in the kidneys and were further enhanced by *BCAA administration*. However, BCKAs were downregulated under diabetic conditions with or without BCAAs supplementation, indicating a defect in BCAA catabolism. Metabolomic analysis revealed crucial issues associated with BCAA metabolism by identifying the pathological network of metabolic profiles in different tissues.

BCAAs in the retina originate from circulation and transportation. To clarify the mechanism by which BCAAs regulate DR development, we first evaluated the levels of BCAAs and BCKAs in the retinas of *db/db* mice and in Müller cells exposed to hyperglycemia by MS detection. In this study, a human retinal Müller cell line-MIO-M1 cell was used in vitro experiments. However, original human cells have not been used. Unsurprisingly, the levels of BCAAs, including leucine, isoleucine, and valine, and BCKAs were significantly increased in the retina and Müller cells under DR conditions. Feeding mice with BCAAs or supplementation in vitro further aggravated the accumulation of BCAAs and BCKAs. The upregulation of BCAAs and their defective catabolism in various tissues has been proposed to be mainly due to the altered enzymatic activity of BCATs and BCKD [28]. The activity of BCATs, the enzyme responsible for BCAA transamination to BCKA, was inhibited under diabetic conditions and was further suppressed by BCAA addition. Subsequently, BCKAs are oxidized by the BCKD complex, which is inhibited by BCKDK. As revealed by protein quantification, BCKDHA and BCKDHB were downregulated in diabetic retinas and hyperglycemia-induced Müller cells, whereas BCKDK levels increased. When treated with a high dose of BCAAs, the downregulation of BCKDHA and BCKDHB was aggravated and the increase in BCKDK was elevated. This confirmed that the increased levels of BCAAs in the retina and Müller cells under high glucose conditions were due to reduced BCAT expression and decreased BCKD complex activity via increased BCKDK expression. Accordingly, the first and second steps of BCAA catabolism are impeded in DR. Our results are consistent with the findings in the muscle and liver in obesity and IR in T2DM, which are the main tissues in which BCAA oxidation occurs, contributing to plasma BCAA levels [28]. In particular, we found that defective BCAA catabolism in the retina under diabetic conditions was exacerbated by BCAA accumulation.

Furthermore, BCAAs activate mTOR signaling, although the mechanisms underlying their effects have not yet been fully elucidated [29]. Increased plasma BCAA levels can lead to persistent mTOR activation via S6 kinase (p70 S6K) [30]. Additionally, BCAA-induced stimulation of the mTOR/p70 S6K pathway has been demonstrated in rodent studies [31–33] and cell experiments [34, 35]. Our previous study confirmed increased mTOR phosphorylation in diabetic retinas [16]. In the present study, we further revealed that mTOR activation occurred through the phosphorylation of p70 S6K and S6K in the retinas of *db/db* mice and hyperglycemic Müller cells. Furthermore, downstream molecules, including those involved in inflammation (TNF-α), angiogenesis (VEGFA), and Müller cell activation (GS and GFAP), were stimulated. Compared to diabetic conditions, the phosphorylation of proteins involved in the mTOR signaling pathway in vivo and in vitro was higher in diabetic mice treated with exogenous BCAAs. Subsequently, the inflammatory response, angiogenic activity, and Müller cell activation were further promoted. The flow cytometry results showed that the apoptosis of Müller cells under hyperglycemic conditions was increased and promoted by upregulated BCAAs. This could be explained by activation of the mTOR signaling pathway, which increases retinal cell apoptosis under DR conditions [36]. Mitochondrial dysfunction is closely associated with DR development [37, 38]. Therefore, in this study, mitochondrial health was determined by quantifying the mitochondrial respiration rate (Seahorse) and ATP production rate. In high glucose–induced Müller cells, mitochondrial respiration was impaired, and mitochondrial biogenesis was also impeded. Furthermore, HG significantly inhibited mitochondrial respiratory capacity and ATP production in Müller cells. Additionally, BCAA accumulation exacerbates impaired mitochondrial respiration and decreases mitochondrial biogenesis in Müller cells under high glucose conditions. Under disease-related stress, damaged mitochondria accelerate apoptosis [39, 40]. Therefore, mitochondrial dysfunction may also deteriorate apoptotic Müller cells under hyperglycemic conditions. It has been reported that mTORC1 controls mitochondrial activity and biogenesis through 4E-BP-dependent translational regulation [41], which suggests that impaired mitochondria in Müller cells resulting from hyperglycemia and BCAAs may be mediated by mTORC1 activation. Although the three BCAAs are normally consumed together and share similar catabolic processes, most studies have explored the physiological roles of BCAAs in combination rather than individually. Growing evidence suggests that BCAAs may have distinct effects on molecular processes and metabolism and may affect the underlying process of various diseases [42]. Identifying the predominant BCAA that activates mTORC1 signaling and affects Müller cells in DR is highly important.

To determine the specific role of individual BCAAs in stimulating the mTORC1 signaling pathway in DR, we used a new high-glucose medium to which each BCAA was individually added. The results showed that BCAA deprivation suppressed the phosphorylation of p70 S6K and S6K in hyperglycemic Müller cells. However, leucine distinctly increased p-p70 S6K/p70 S6K and p-S6K/S6K in Müller cells exposed to high glucose without complicated BCAAs. This stimulation was greater than that in the high-glucose medium containing BCAAs. We also observed that isoleucine induced the activation of the mTORC1 signaling pathway compared to that in hyperglycemic medium without BCAAs. However, valine did not significantly increase the phosphorylation of the mTORC1 complex. These results suggest that leucine is the main BCAA that induces mTORC1 activation in Müller cells under DR conditions. The mechanism by which the mTORC1 pathway senses leucine remains debatable. Growing evidence indicates that leucine-binding proteins, such as sestrins, serve as leucine sensors in various facets of organismal function [43–45]. In cultured cells, Sestrin2 suppresses mTORC1 by interacting with and inhibiting the GTPase-activating protein 2 (GATOR2) complex, which positively regulates mTORC1 signaling. Under conditions of abundant leucine, Sestrin2 can bind to it and be released from its complex with GATOR2, thus activating mTORC1 signaling [43]. Another in vivo study showed that leucine-induced activation of mTORC1 in skeletal muscles occurred primarily through the release of Sestrin1 from GATOR2 [46]. Sestrin1 and Sestrin2 have been identified as leucine sensors in the mTORC1 pathway in mouse tissues in vivo [44]. In contrast to Sestrin2, LeuRS, an intracellular leucine sensor, initiates mTORC1 activation [47]. To investigate whether the modulation of mTORC1 by dietary leucine requires Sestrin1 or Sestrin2 and the coordination of sestrins and LeuRS, we generated Müller cells with knockdown of Sestrin1, Sestrin2, or LeuRS. Under Sestrin2-downregulated conditions, high levels of leucine did not induce the activation of mTORC1 signaling. Although interference with Sestrin1 decreased leucine-induced mTORC1 stimulation, the decrease was not statistically significant. Therefore, in hyperglycemic Müller cells, leucine activates mTORC1 signaling mainly through Sestrin2 binding. Additionally, LeuRS knockdown significantly inhibited the phosphorylation of p70 S6K and S6K in Müller cells with the addition of leucine under high glucose conditions. This suggests that Sestrin2 and LeuRS coordinate with each other to regulate mTORC1 signaling in response to leucine in Müller cells under in vitro DR conditions. Targeting Sestrin2 significantly ameliorated impaired cell viability caused by leucine overload in Müller cells exposed to high glucose. In the future work, it may be of significance to employ targeting Sestrin2 in vivo in*db/db*mice to explore the protective effects on ameliorating DR development. By vitreous injection of anti- Sestrin2 may be a potential therapy to protect early stage of DR to develop into neovascularization.

## Conclusions

Our previous work found that elevated levels of BCAAs were observed in vitreous and aqueous humor of patients with DR, which hinted us to further investigate the mechanisms of BCAA accumulation in DR. The present study demonstrated that impaired BCAA catabolism was responsible for BCAA accumulation in the retina and Müller cells under DR conditions, which was exacerbated by BCAA administration. Leucine was the main BCAA that activated mTORC1 signaling by interacting with Sestrin2 and LeuRS in Müller cells under high glucose conditions. Future work exploring targeting Sestrin2 to reduce the toxic effects of BCAA accumulation in vivo in *db/db* mice should be addressed. Targeting Sestrin2 may protect Müller cells during DR development by inhibiting mTORC1 activation and subsequent pathophysiological reactions. These studies would help develop a therapeutic strategy targeting Sestrin2 to protect the early stage of DR and postpone DR development with angiogenesis and neurodegeneration.

## Data Availability

The datasets used and/or analysed during the current study are available from the corresponding author on reasonable request.

## Abbreviations

BCAAs: BranchEd-Chain Amino Acids
BCAT: Branched-Chain Aminotransferase
BCKAs: Branched-Chain α-Keto Acids
CCK-8: Cell Counting Kit-8
DR: Diabetic Retinopathy
DKD: Diabetic Kidney Disease
GATOR2: GTPase-Activating Protein 2
IR: Insulin Resistance
KIC: α-Ketoisovalerate
KIV: α-Ketoisobutyrate
KMV: α-Keto-β-Methyl Valeric Acid
LC‒MS: Liquid Chromatography‒mass spectrometry
LeuRS: Leucyl-Trna Synthetase
mTORC1: Mechanistic Target of Rapamycin Complex 1
OCR: Oxygen Consumption Rate
siRNA: Small Interfering RNA
T2DM: Type 2 Diabetes Mellitus
WT: Wild-Type
